# Circuits and Synapses: Hypothesis, Observation, Controversy and Serendipity – An Opinion Piece

**DOI:** 10.3389/fncir.2021.732315

**Published:** 2021-09-16

**Authors:** Alex M. Thomson

**Affiliations:** Department of Pharmacology, UCL School of Pharmacy, London, United Kingdom

**Keywords:** neurone, axon, dendrite, neocortex, NMDA, transmitter, history

## Abstract

More than a century of dedicated research has resulted in what we now know, and what we think we know, about synapses and neural circuits. This piece asks to what extent some of the major advances – both theoretical and practical – have resulted from carefully considered theory, or experimental design: endeavors that aim to address a question, or to refute an existing hypothesis. It also, however, addresses the important part that serendipity and chance have played. There are cases where hypothesis driven research has resulted in important progress. There are also examples where a hypothesis, a model, or even an experimental approach – particularly one that seems to provide welcome simplification – has become so popular that it becomes dogma and stifles advance in other directions. The nervous system rejoices in complexity, which should neither be ignored, nor run from. The emergence of testable “rules” that can simplify our understanding of neuronal circuits has required the collection of large amounts of data that were difficult to obtain. And although those collecting these data have been criticized for not advancing hypotheses while they were “collecting butterflies,” the beauty of the butterflies always enticed us toward further exploration.

## The “Neurone Doctrine” *Versus* the “Reticular Concept”

Cajal’s description of his first sight of cerebral cortex stained with Golgi’s method is explanation enough for his dedicating the rest of his life to the study of the nervous system:

‘*Against a clear background stood black threadlets, some slender and smooth, some thick and thorny, in a pattern punctuated by small dense spots, stellate or fusiform. All was sharp as a sketch with Chinese ink on transparent Japan-article. And to think that that was the same tissue which when stained with carmine or logwood left the eye in a tangled thicket where sight may stare and grope for ever fruitlessly, baffled in its effort to unravel confusion and lost for ever in a twilit doubt. Here, on the contrary, all was clear and plain as a diagram. A look was enough. Dumbfounded, I could not take my eye from the microscope.*’ (Sherrington, 1935, quoting Cajal, 1899-1904; Mazzarello, 1999)

A number of scientists starting with Hooke, 1665 are credited with contributions to the theory that living organisms comprise one or more cells; that the cell is the basic unit of life; and that cells are derived from pre-existing cells. In [Bibr B129][Bibr B129] extended the theory from plants to animals and – based on the work of Santiago Ramón y Cajal and many others – His (1887) and Forel (1887) applied it to neurones – the neurone doctrine. However, when Cajal began to study the structure of the nervous system in 1887, the central nervous system (cns) was widely believed to comprise a diffuse protoplasmic network or reticulum (von Gerlach, 1871).

Nearly 20 years later, On November 11, 1906, Golgi (1843-1926), who had still not abandoned the Reticular Hypothesis, began his Nobel Lecture (Golgi, 1906) entitled **“The neuron doctrine – theory and facts**” – with gloves off:

‘*It may seem strange that, since I have always been opposed to the neuron theory – although acknowledging that its starting-point is to be found in my own work – I have chosen this question of the neuron as the subject of my lecture, and that it comes at a time when this doctrine is generally recognized to be going out of favour*.’

‘*At a point of time that the results of black staining had hardly started to become generally known, while I had already for about 10 years achieved results much better in terms of clarity than those which had attracted attention elsewhere, the idea that cells and nerve cells formed an anatomical unit became much more acceptable to the mind in a far more objective way than that made possible by previous studies*.’

Golgi then introduced the ideas upon which the neurone theory – as formulated by Waldeyer (1891) – is based: that the neurone is an embryological unit; that the neurone – even in the adult – is one cell; and that the neurone is a physiological unit. He then continues:

‘… *From the physiological point of view, the neuron theory finds its most perfect expression in the so-called theory of dynamic polarization, which van Gehuchten (1891, 1908) had already outlined, and which my distinguished colleague Ramón y Cajal developed and supported in a most complete manner*.’

‘*The fundamental points of the doctrine may be summed up like this: the transmission of nerve impulses is conducted from the protoplasmic extensions* [dendrites] *and the cell body towards the nerve extension* [axon]; *consequently, each nerve cell possesses a receiving apparatus constituted by the body and the protoplasmic processes, a conducting apparatus – the nerve process – and a transmitting or discharging organ. The protoplasmic processes should, therefore, act as conductors towards the cell body, and the nerve process should act as a conductor away from it.****The author himself has successively, as one knows successively modified his theory***, *either for adapting it to some particular topographical arrangements of the point of origin of the nerve process, or to harmonize it with data resulting from more intensive studies…*.’

‘…*I shall therefore confine myself to saying that, while I admire the brilliancy of the doctrine which is a worthy product of the high intellect of my illustrious Spanish colleague, I cannot agree with him on some points of an anatomical nature which are, for the theory, of fundamental importance*…’

Before turning to Cajal’s defence of the neurone doctrine, it is worth noting that Cajal would not – at least not as quickly – have made the observations summarized in his own lecture without Golgi’s silver staining method, or “black reaction” (first publicized in 1873) – one that was severely criticized for many years as being unreliable. The method is unreliable – fortunately – since were it to stain every neurone in a slab of tissue equally, it would be practically impossible to distinguish any structure within it. It is also conceivable that Golgi would not have shared the Nobel Prize for Medicine or Physiology in 1906 had Cajal not used his method so successfully.

Cajal (1906) began his Nobel Lecture, **“The structure and connexions of neurons**,” the next day, by summarizing his early work:

‘*We applied Golgi’s method, firstly in the cerebellum… and so on of embryos and young animals, and our observations revealed, in my opinion, the terminal arrangement of the nerve fibres. These fibres, ramifying several times, always proceed towards the neuronal body, or towards the protoplasmic expansions around which arise plexuses of very tightly bound and rich nerve nests. The pericellular baskets and the climbing plexuses, and other morphological structures, whose form varies according to the nerve centres being studied, confirm that the nerve elements possess reciprocal relationships in contiguity but not in continuity. It is confirmed also that those more or less intimate contacts are always established, not between the nerve arborizations alone, but between these ramifications on the one hand, and the body and protoplasmic processes on the other. A granular cement, or special conducting substance would serve to keep the neuron surfaces very intimately in contact*.’ [One of many perspicacious predictions made by Cajal]

‘*From the whole of these facts, the neuronal doctrine of His and of Forel, accepted by many neurologists and physiologists, is derived as an inevitable postulate. However, it must be said that some of the physiological inferences drawn… have been contended, and naturally cannot be considered as unimpeachable dogmas.* ***Present-day science… has not the right to foretell the future****… We cannot reject, a priori, the possibility that the inextricable forest of the brain, the last branches and leaves of which we imagine ourselves to have discerned, does not still possess some enigmatic system of filaments binding the neuronal whole, as creepers attach the trees of tropical forests. This is an idea which, appearing to us with the prestige of unity and of simplicity, has exerted and still exerts, a powerful attraction for even the most serene of spirits. True, it would be very convenient and very economical from the point of view of analytical effort if all the nerve centres were made up of a continuous intermediary network between the motor nerves and the sensitive and sensory nerves.* ***Unfortunately, nature seems unaware of our intellectual need for convenience and unity, and very often takes delight in complication and diversity***.’

Cajal could, however, also be dogmatic and was not consistently generous to Golgi and the other “anti-neuronists”:

‘*… The irresistible suggestion of the reticular conception… (the form of which changes every 5 or 6 years) has led several physiologists and zoologists to object to the* [neurone] *doctrine… All* ***their allegations are based on the findings by incomplete methods*** *showing far less than those which have served to build the imposing edifice of the neuronal conception*.’

‘*… my recent researches…* ***done with a more revealing process than those used by the anti-neuronists***, *proves in the most incontrovertible fashion the lack of foundation for the hypothesis of the discontinuous development of nerve fibres*.’

**‘*Like many scientific errors professed in good faith by distinguished scientists****the link theory is the result of two conditions: one subjective, and the other objective*. ***The first is the regrettable but inevitable tendency of certain impatient minds, to reject the use of elective methods***, *such as those of Golgi and of Ehrlich****which do not lend themselves easily to improvisation****;****the second is the exclusive application of processes simple and convenient***…’

Cajal also has a word of caution for those who criticize without sufficient foundation:

‘*To sum up: from the entirety of the observations which we have just shown, and from many others… the doctrine of neurogenesis of His is clearly revealed as an inevitable postulate*. ***We mourn this scientist who***, *in the last years of a life so well filled*, ***suffered the injustice of seeing a phalanx of young experimenters treat his most elegant and original discoveries as errors***.’ Wilhelm His, who might conceivably have shared the prize had he lived, had died in 1904 (See also López-Muñoz et al. (2006); Shepherd (2016).

Interestingly, neither Golgi nor Cajal mentions the concept of the “synapse” as introduced by Charles Sherrington (1897, 1906), though it supports the neurone doctrine. Students are recommended to read and learn from these articles. Sherringtom did not call his ideas hypotheses, or doctrines, he simply followed a train of thought founded upon his own ground-breaking work and his understanding of the literature. His insight was remarkable – if his arguments a little verbose:

‘*…If we assume, and we seem to be entitled to assume, that each filament arising as a division either of a dendrite, or an axon may serve as the path of a separate nerve impulse, the constitution of the grey matter may be regarded as offering opportunities for the most complex dispersion of nervous impulses, that the impulse as it passes… into the dendrite of another cell, starts… an impulse having characteristics different from its own…*’

‘*…The ganglion of the sympathetic system is somewhat more complex since we have reason to think that the cells may have much branched dendritic processes and that a nerve fibre entering a ganglion may end by* ***synapsis*** *with one of the cells. But even this falls far short of the complexity of the arrangements in the spinal cord…*’

‘*The distinguishing feature of the spinal cord is the extreme complexity in which the processes, dendrites and axons, of its constituent cells are arranged; and we may infer that the special powers of the cord are in large measure dependent on this complexity. We may suppose, and indeed we probably must suppose, that what takes place in a particular cell is determined also by the intrinsic nature of the cell… for instance two impulses of a like nature impinging on two cells by means of similar* ***synapses*** *may produce in one cell an event of one kind, and in the other an event of another kind (Foster and Sherrington (1897).*’

‘*Nowhere in physiology does the cell-theory reveal its presence more frequently in the very framework of the argument, than at the present time in the study of nervous reactions.*’

‘*…If there exists any surface or separation at the nexus between neurone and neurone, much of what is characteristic of the conduction exhibited by the reflex arc might be more easily explicable. At the nexus between cells if there be not actual confluence, there must be a surface of separation. At the nexus between efferent neurone and the muscle-cell, electrical organ, etc., which it innervates, it is generally admitted that there is not actual confluence of the two cells together, but that a surface separates them; and a surface of separation is physically a membrane…*’

‘*… in the neurone-chains of the gray-centred system of vertebrates histology on the whole furnishes evidence that a surface of separation does exist between neurone and neurone. And the evidence of Wallerian secondary degeneration is clear in showing that that process observes strictly a boundary between neurone and neurone and does not transgress it. It seems therefore likely that the nexus between neurone and neurone in the reflex-arc, at least in the spinal arc of the vertebrate, involves a surface of separation between neurone and neurone…*

‘*In view, therefore, of the probable importance physiologically of this mode of nexus between neurone and neurone it is convenient to have a term for it. **The term**” **introduced has been synapse.^170^” (**Sherrington, 1906, citing Foster and Sherrington, 1897).*’

Nor did either Cajal or Golgi cite Sherrington’s demonstration of antidromic conduction (1897) which might be interpreted as contradicting the theory of dynamic polarization. However, while not an attractive habit, not citing those who provide evidence that conflicts with your own, or those who might be credited with an earlier discovery or hypothesis, is not unique: Sherrington had not cited William James (1890) who proposed that nervous pathways always run one way – from sensory cells to motor cells. As science becomes more competitive (a horrible concept to apply to the pursuit of knowledge) and grant awarding agencies and “high impact” journals demand “originality,” some of our peers become increasingly disinclined to give due credit (unless they think one of the authors will be reviewing their article or application).

## Dale’s Hypothesis

“Dale’s hypothesis” is another well known and popular example of a statement that is cited frequently as a “law” [though admittedly it is more commonly called a law in theoretical and psychological, than in physiological or pharmacological publications]. This “law” is supposed to state that every axon terminal of a given neurone releases only one and the same neurotransmitter.

We should, however, look first at what Sir Henry Dale actually wrote:

“*It is to be noted, further, that* ***in the cases for which direct evidence is already available***, *the phenomena of regeneration* ***appear to indicate*** *that the nature of the chemical function, whether cholinergic or adrenergic, is characteristic for each particular neurone, and unchangeable. When we are dealing with two different endings of the same sensory neurone, the one peripheral and concerned with vasodilatation and the other at a central synapse*, ***can we suppose*** *that-the discovery and identification of a chemical transmitter of axon-reflex vasodilatation would* ***furnish a hint*** *as to the nature of the transmission process at a central synapse? The possibility has* ***at least some value as a stimulus to further experiment***.” (Dale, 1934)

Dale made a supposition that could be invalidated by experiment; one that could act as a stimulus to further experimentation. One wonders whether Dale had the opportunity to read Karl Popper’s “*Logik der Forschung*” (1933–4) circulating as a typescript at that time. We will return to Popper soon.

## Electrical or Chemical Transmission?

Dale’s work was central to the identification of pharmacological tools that could aid the investigation of synaptic transmission. It is perhaps unfortunate that the tools then available were largely limited to those that affect cholinergic and adrenergic transmission. For example, John (Jack) Eccles challenged the ventral root potentials elicited by dorsal root volleys in the hemisected spinal cord of the frog with acetylcholine (ACh) and prostigmine (a cholinesterase inhibitor) *in vitro*. On obtaining negative results, he concluded: “*these observations refute the hypothesis that ACh mediates synaptic transmission in the frog’s spinal cord*.” And, assuming that his results constituted evidence against *any* chemical transmission in the spinal cord, he went further: “*The present observations likewise accord with the electrical hypothesis of transmission..*.”

Eccles was soon propounding electrical hypotheses for both central excitation (Eccles, 1945) and inhibition (Brooks and Eccles, 1947; Eccles, 1947). For nearly two decades Eccles argued that synaptic events were not mediated by chemical, but by electrical transmission. By the early 1950’s, however, he had accepted that synaptic transmission was chemically mediated – at some synapses: first at the motor end-plate, then in several autonomic ganglia and their end organs. During this period, chemical transmission and the extraordinary work done by some of our most talented scientists were frequently relegated to the periphery – in more ways than one. In was, however, Eccles et al. (1954) who finally demonstrated that the axons of motoneurones, which release acetylcholine at the motor end-plate, also release acetylcholine from their collaterals to excite Renshaw cells in the spinal cord. Dale had not raised his supposition to the level of a “principle.” It was Eccles who coined the term “Dale’s Principle,” in that article.

Eccles had not, however, accepted that central synaptic *inhibition* was chemically mediated – not yet. Since, acetylcholine and “a substance related to adrenaline” were the only accepted neurotransmitters in mammals, to refute the suggestion that central inhibition was chemically mediated it was sufficient to demonstrate that neither was responsible for the reflex inhibitions so elegantly demonstrated by Eccles’ mentor, Sir Charles Sherrington. Moreover, a number of respected and influential scientists agreed with him.

## Karl Raimund Popper and Hypothesis-Driven Research

An announcement from the Physiological Society for this year’s Rob Clarke Awards calls for project proposals that include a hypothesis. It is not at all clear what they mean by this. Let us turn to Popper who is often cited as the originator of the concept of “hypothesis driven research.” In many ways he was, but he was very precise in what he meant by it. He most certainly did not favor investigations undertaken to provide support for a hypothesis. Popper decried the enthusiasm his friends displayed for the theories propounded by Marx, Freud and Adler because they ‘*appear to be able to explain practically everything that happened within the fields to which they referred*.’ While his friends thought this constituted the strongest evidence in favor of these theories, Popper believed it was their greatest weakness. His preference was for theories that could – like Einstein’s “Theory of Relativity” – be refuted by a single observation; an observation that was well within the capabilities of astronomers at that time (given a convenient eclipse): ‘… *the impressive thing about this case is the risk involved in a prediction of this kind. If observation shows that the predicted effect is definitely absent, then the theory is simply refuted.*’

Popper later reformulated the ideas he had first considered during the winter of 1919-20 in seven points, summarizing them thus: ‘… *the scientific status of a theory is its falsifiability, or refutability, or testability*.’ In brief, Karl Popper’s first four points state that confirmations or verifications for nearly every theory can be found – if we look for them, but that a “verification” should only be accepted if the expected result would have refuted the theory. A theory that could not be refuted by a feasible and predictable observation or experiment is therefore “unscientific.” One wonders how many of the theories pertaining, for example, to the basis of consciousness, that aging scientists sometimes like to propose (safe in the knowledge that they will not be refuted in their life-time), theories that will ensure lasting fame if supported, would be deemed “unscientific” by Popper.

In the preface to his elegant monograph Mircea Steriade, an equally fierce critic and loyal friend to those lucky enough to know him, wrote: ‘*The evolution of work on extremely simplified preparations has reached the point where some investigators are not shy to jump from single-cell properties to global brain functions, such as arousal and sleep, paroxysmal events of the epileptic type, and even conscious thinking*’ (Steriade, 2001).

It appears to have been Popper’s ideas that encouraged his life-long friend, John Eccles, to continue to propose hypotheses and to challenge the alternatives: “*From 1945 onward I was deeply under the influence of Karl Popper, who stressed the necessity to formulate clear hypotheses and then test them by rigorous experiment.*” (Eccles, 1982). Sir John Eccles was possibly more often and more spectacularly wrong than any life scientist before or since. However, along the way he collected an impressive selection of honors, achieved a *tour de force* with the first intracellular recordings from central mammalian neurones, provided evidence in favor of chemical transmission at central excitatory synapses, and finally refuted his own electrical “Golgi-cell hypothesis” (Brooks and Eccles, 1947) of central synaptic inhibition (Brock et al., 1952a,b).

Old habits die hard, however, and, having refuted one hypothesis, the authors immediately postulated another, perhaps equally unlikely hypothesis: ‘*Since it may be assumed that the resting motoneurone is in a steady state for potassium and chloride, any alteration in the permeability to these ions would have no effect on the membrane potential (Hodgkin, 1951). It would thus appear that the simplest explanation of the hyperpolarization produced by an inhibitory transmitter substance would be that it caused a net outward flux of sodium ions, which most probably is attributable to stimulation of the sodium pump*.’

Refuting this hypothesis with the most obvious experiment – blocking the sodium pump – would have posed significant problems, both practical and in terms of interpretation. This new hypothesis could however, have been challenged by further reference to the article by Hodgkin, which he cites (Hodgkin, 1951): ‘*Katz & I used the constant field theory of Goldman (1943) to calculate the resting potential of Lolio (Hodgkin and Katz, 1949)… Other evidence indicated that relative value of the permeability coefficients was approximately: P_*K*_ : P_*Na*_ : P_*Cl*_ = 1 : 0.04 : 0.45, and a theoretical resting potential of 59.5 mV. was obtained on this basis*.’

Although he fought his electrical corner long and hard, Eccles was prepared to put his own ideas to the test (or at least to try to refute alternative hypotheses) and, when the evidence became overwhelming, to accept that alternative (Eccles, 1982). Not everyone is prepared to put their own hypotheses to the test. Eccles must, however, have enjoyed watching the accumulation of evidence in favor of gap junctions between neurones. In 1982 he wrote: ‘… *the rejection of electrical transmission was correct for all synapses in the controversy, but electrical transmission has made a “come back,” even in the vertebrate CNS, with the recognition of a special type of synapse, the gap junction.*’ By the time he died in 1997 connexins had been identified in the brain, and their involvement in brain development (e.g., Nadarajah et al., 1997) and synchronization of neuroendocrine cells in parturition and lactation (Micevych et al., 1996) had been proposed (Bennett, 1997; Bennett and Pereda, 2006, for commentary).

Popper’s fifth and sixth points largely expand upon the first four, but his final point is worth repeating in its entirety:

*7. Some genuinely testable theories, when found to be false, are still upheld by their admirers – for example by introducing ad hoc some auxiliary assumption, or by reinterpreting the theory ad hoc in such a way that it escapes refutation. Such a procedure is always possible, but it rescues the theory from refutation only at the price of destroying, or at least lowering, its scientific status.*

Dale’s supposition is an example. When evidence began to emerge that some neurones release more than one transmitter, the various statements derived from Dale’s original supposition required only the addition of “*or substances*” to encompass the new data. It was, however, a long time before some quite eminent scientists accepted the evidence that, although they released the same transmitter(s), not all terminals of a single axon behave in the same way in other respects. However, once they had seen the evidence in their own labs, they became new converts, happy to claim what had once been heresy, as their own.

## A Historical Digression

In the 1930’s Germany’s loss became Britain’s gain, with outstanding scientists like Marthe Vogt, Heinz Schild and Wilhelm Feldberg who came to London to work with Dale at the National Institute for Medical Research, Edith Bülbring, who joined Joshua Burn’s department at the Pharmaceutical Society of Great Britain (soon to become The School of Pharmacy) and Hermann (Hugh) Blaschko who joined AV Hill at University College London. Sir Henry Dale had inspired many physiologists to study synapses and amongst them several émigrés. Perhaps particularly worthy of our gratitude is Bernard Katz who also joined AV Hill, in 1935. Of his mentor and the time he spent working with him, Katz wrote:

‘*It was an outstanding piece of good luck to have been taken on as an apprentice to A.V. Hill; it was the decisive influence on my life and career… He was the person from whom I have learned more than anyone else, about science and about human conduct… A.V. Hill was the most naturally upright man I have ever known… To be associated with a man of his stature at a formative period of one’s life is indeed a great gift of fortune*’ (Katz, 1978).

Many who had the opportunity to know Bernard Katz would say the same of him. One brief story exemplifies both his humility and his pleasure in accepting an intellectual challenge. In his retirement, UCL provided him with a word processor on which he was expected to write his memoirs. This task, it appears, did not appeal, but in a shop in nearby Tottenham Court Road he found a program that would play chess on the word processor (chess had occupied many hours during his student days). Sir Bernard was not seen at coffee for several days. Locked away in his office he tackled the question: how many future moves could the program compute? Having solved that problem – only eight – he could beat it every time and returned, reluctantly, to the lecture he was to give to the Physiological Society (Katz, 1986, recommended reading).

Also typical, was the way in which he began his history of neuroscience in biography, for the Society for Neuroscience some 10 years later (Katz, 1996):

‘*In October 1924 my great friend and teacher, A.V. Hill, made his first visit to America… The first evening after his arrival… he gave a public lecture… on “The Mechanism of the Muscle.” At the end of his talk, a serious-looking elderly member of the audience got up and asked disapprovingly what practical use the speaker thought there was in his research. Professor Hill considered for a moment whether he should enumerate the many cases in which immense and obvious benefit to humankind had come from discoveries and experiments that were made purely to satisfy the intellectual curiosity of the investigator, but now I let him tell his own story:*

*“To prove to an indignant questioner on the spur of the moment that the work I do was useful seemed a thankless task and I gave it up. I turned to him with a smile and finished*, ***”To tell you the truth we don’t do it because it is useful but because it’s amusing****.” The answer was thought of and given in a moment: it came from deep down in my soul… the newspapers next day… came out with headlines “Scientist Does It Because It’s Amusing!” And if that is not the best reason why a scientist should do his work, I want to know what is…*. ***With this faith in the ultimate usefulness of all real knowledge a man may proceed to devote himself to a study of first causes without apology, and without hope of immediate return.”***

In the prewar years Katz also collaborated with Alan Hodgkin and Andrew Huxley at the Plymouth Marine Laboratory, to study action potential (AP) propagation and neuromuscular transmission in invertebrates (eg. Katz, 1936, 1949). Just before and during the early months of the war he worked on neuromuscular transmission with Stephen Kuffler in Sydney, before joining the Royal Australian Air Force, as a radar officer. Radar introduced him to the most up to date electronics, an advantage shared by Andrew Huxley [radar control of anti-aircraft guns for British Anti-Aircraft Command] and Alan Hodgkin [airborne centimetric radar system for Royal Aircraft Establishment]. This and the sometimes unofficial acquisition of military supplies, were to prove critical in future developments. Tiny currents, or changes in potential could be amplified, displayed on a cathode ray oscilloscope and photographed, instead, for example, of firing a smoked glass slide past a needle attached to a galvanometer to record action currents. It is worth noting that most notable physiologists were also competent-, some often inspired, engineers.

Katz returned to UCL in 1946, where, in the summer of 1948 Paul Fatt joined him. Armed with new technology and glass microelectrodes (Graham and Gerard, 1946; Hodgkin and Nastuk, 1949; Ling and Gerard, 1949) they began the now legendary intracellular recordings from the motor end-plate. Young scientists could still learn a great deal from their meticulous analysis of the limitations of their technique and equipment and the efforts they made to understand and mitigate them (Fatt and Katz, 1951). It was undoubtedly this attention to detail that allowed them to make – and to convince the scientific community of – the discovery that was to change our understanding of synaptic transmission for ever.

## Hypothesis-Driven or Observation?

Thus far, Fatt and Katz (1950a) had extended existing knowledge by determining the electric charge that passes through the end-plate membrane during the transmission of one impulse and had thrown some light on the mechanism by which this transfer of ions is brought about. However, no as yet formulated hypothesis could have inspired them to drop what they were doing to investigate “biological noise” (Fatt and Katz, 1950b, 1951).

The 1952 article* (Fatt and Katz, 1952) suggests no theoretical basis upon which the experiments might have been based; no suggestion is made that the outcome could have been predicted from any existing knowledge or theory. The brief introduction simply reads: “***The present study arose from the chance observation*** *that end-plates of resting muscle fibres are the seat of spontaneous electric discharges which have the character of miniature end-plate potentials. The occurrence of spontaneous subthreshold activity at an apparently normal synapse is of some general interest, and a full description will be given here of observations which have been briefly reported elsewhere*.”

*Note: It may be of interest to younger readers that at that time, a publication in “Nature” was seen as a preliminary report, the limited space available for the description of methods was particularly decried. If a finding was to be taken seriously, the rapid publication in “Nature” had to be followed by a full account in a more “respectable” journal. Otherwise, it was assumed to have been an incomplete, or unrepeatable result.

Katz had observed events like these miniature end-plate potentials (m.e.p.p.s) in Kuffler’s laboratory while recording from the surface of isolated muscle fibers, but, suspecting that they resulted from injury discharge at the nerve endings, paid little attention. This underlines the point made earlier. Confidence in your technique is vital, and not because you have acquired the latest, shiniest piece of equipment or software, or followed a protocol recently described by a giant in your field, but because you have tested the ability of your system to record biological events faithfully, under conditions that you and your peers can understand – and reproduce.

## The “Quantal Hypothesis”

del Castillo and Katz (1953) and Boyd and Martin (1956) applied the “Quantal Hypothesis” to the neuromuscular junction and then to spinal motoneurones (Katz and Miledi, 1962, 1963), attracting a number of suggestions as to the structural elements that might underlie the release of discrete packages of transmitter – “quanta”, but physiologists had to wait for electron microscopists to reveal the membrane-bound vesicles clustered close to the presynaptic membrane in a wide variety of axon terminals – and to recognize them for what they were (Palade and Palay, 1954, 1955; de Robertis and Bennett, 1955; Palay, 1956; Birks et al., 1960, and many others). One is reminded of Ted Jones, friend and support to many, describing the informal meetings in Oxford’s Department of Human Anatomy in the 1960s, when recently printed micrographs were passed around and ideas about what on earth they might be looking at were discussed. Incidently, these early ultrastructural studies also demonstrated that one of arguments against chemical transmission – that the synaptic cleft was too wide to account for the brief delays recorded – had been due to the failure of silver impregnation techniques to label the entire presynaptic terminal (Gray and Guillery, 1961). The synaptic cleft was not 1 μm wide – as silver staining of neurofibrils observed with the light microscope suggested – but some 30 to 50 times narrower – only 200-300Å in diameter.

It was not hypothesis-driven research that had resulted in the discovery of miniature end-plate potentials, or of the vesicles that released the transmitter. It was observation, with an eye trained to recognize what might be important, followed by meticulous experimentation to explore the properties of what had been observed. Chance had brought together those who had the theoretical and technical expertise needed, when the equipment required to study the phenomenon in sufficient detail was available and at a time and in places where exceptional minds were at work.

## Influential Hypotheses Can Result From Serendipitous Observations, but Nevertheless Results in Fiercely Defended Dogma

While physiologists and pharmacologists were exploring synaptic transmission *in vitro*, physiologists working *in vivo* were discovering how elegantly the different parts of the body and different somatosensory modalities were represented in the somatosensory cortex (Mountcastle et al., 1955, 1957). In 1957 Vernon Mountcastle wrote: “*These data… support an hypothesis of the functional organization of this cortical area. This is that the neurons which lie in narrow vertical columns, or cylinders, extending from layer II through layer VI make up an elementary unit of organization, for they are activated by stimulation of the same single class of peripheral receptors, from almost identical peripheral receptive fields, at latencies which are not significantly different for the cells of the various layers*.”

Inspired by this work and building upon the pioneering studies of Barlow (e.g., Barlow et al., 1957) in retinal ganglion cells, and Hubel’s preliminary studies in the lateral geniculate nucleus (LGN), David Hubel and Torsten Wiesel began investigating the receptive field properties of neurones in striate cortex (Hubel and Wiesel, 1959). Just as the somatosensory cortex contained a somatic map, visual area V1, appeared to contain a map of the retina. The cortical cells were not, however, very responsive to stimuli that would have activated retinal ganglion, or LGN cells (light and dark spots). It was not until the shadow of the edge of a slide being loaded into the projector happened to pass through the receptive field of a cell near their electrode, that a strong response was elicited. The dark edge was clearly oriented appropriately for that cell. One wonders how many times a slide was loaded, but the cell(s) near the electrode failed to respond because they preferred a different orientation. Serendipity – without which orientation selectivity might not have been discovered – had intervened once again.

In 1962, they presented a simple circuit that could explain the orientation preferences of what they named “simple cells.” They proposed that many lateral geniculate nucleus (LGN) cells – with approximately circular receptive field “on” centers and “off” surrounds that are arranged along a straight line on the retina – deliver excitatory input to a single cortical cell. The resultant receptive field of that cortical cell would be an elongated “on” center, with “off” flanks of the same orientation. In that model, reduced firing, when an inhibitory part of the receptive field is illuminated, results not from inhibition in the cortex, but from cessation of tonic excitation – the inhibition having already occurred at a lower level, in the retina and/or LGN (Hubel and Wiesel, 1962).

This article and many more from the same group made significant contributions to what we know about of receptive fields in the several visual areas. However, the more widely recognized Hubel and Wiesel’s work became, the more necessary it was for anyone hoping to publish recordings in visual cortex to categorize their cells according to Hubel and Wiesel’s criteria – before doing anything else. This was a tall order for intracellular recordings *in vivo* at that time. Hubel and Wiesel did consider a scheme in which direct inhibitory connections replaced the direct excitatory connections in their model (replacing the corresponding LGN neurones with “on” instead of “off” center cells and *vice versa*). However, the simple scheme based solely on cortical excitation was so compelling and deliciously simple that a role for local inhibition in fine tuning was rarely contemplated by any in the field. Despite the demonstrable importance of inhibition in motor control; the exquisite diversity of “short axon neurones” described in neocortex long before by Cajal and many others since; and the growing evidence that these were indeed inhibitory neurones, many still considered cortical inhibition as existing simply to prevent the generation of epileptiform activity.

It was not until a decade later, that Benevento et al. (1972) made intracellular recordings in striate cortex and found that “…*in many cells a striking quantitative change of inhibition was seen when the orientation of the stimulus or the direction of movement was changed*…” This indicated that inhibition is also orientation- and direction-sensitive. Adam Sillito (1975) then demonstrated the effect of this inhibition. The GABA_*A*_ receptor antagonist, Bicuculline, applied iontophoretically modified the receptive field properties of neurones in the cat striate cortex, sometimes dramatically. With GABA_*A*_ receptors blocked, the division of simple cell receptive fields into “on” and “off” regions was lost, orientation selectivity was reduced, while directional sensitivity could even be eliminated.

In his Nobel Lecture Hubel (1981) suggested that inhibitory connections might be necessary to explain direction selectivity in complex cells. He did not, however, cite Benevento et al., or Sillito, but a study of retinal ganglion cells (Barlow and Levick, 1965). Similarly, although he cited Hendrickson et al. (1981) for the periodic pattern of cytochrome oxidase staining in layers II and III, he did not mention that they had also found glutamic acid decarboxylase (GAD) positive axon terminals colocalized with the cytochrome oxidase. Wiesel (1981) in what he did at least call a biased account, did not mention inhibition at all. According to a PubMed search, Benevento’s 1972 article has been cited fifty times and Sillito’s 1975 article 199 times, but not it appears, by Hubel or Wiesel.

No more need be added than to repeat the advice to young academics given by two distinguished historians, Cantor and Schneider (1967):

“*You must be especially careful about historical opinions that are so commonplace that they have been accepted as inviolable truth and inevitable assumption. The more fashionable and cliché ridden an historical judgment, the more traditional a general inference, the more you must be on your guard. Such cliches influence all dependent judgments on a particular era and inhibit a fundamental reconsideration of basic trends and movements.*”

‘*Unfortunately, nature seems unaware of our intellectual need for convenience and unity, and very often takes delight in complication and diversity*’ (Cajal, 1906). If complication and diversity do not delight your soul, it is possible that neuroscience is not the field for you.

Leaping forward in time, but not location, Binzegger et al. (2004) developed a quantitative description of the synaptic circuitry of cat area 17 (V1 equivalent), using Peters’ rule (Peters and Feldman, 1976; Peters and Payne, 1993). They analyzed the elegant three dimensional reconstructions of neurones they had filled with horse radish peroxidase *in vivo*, as well as thalamo-cortical axons of X and Y lateral geniculate nucleus (LGN) relay cells and equivalent data from other groups, and a wealth of published anatomical and statistical data. When compared with estimates based on stereological analysis (Beaulieu and Colonnier, 1985), large discrepancies allowed them to confirm previous findings (e.g., Lund, 1988; Lund et al., 2003; White, 2007; for reviews), that substantial excitatory inputs, from subcortical structures and other cortical regions, provide many of the boutons in layers 4 and 6. For local connections, estimates of those involving each class of potential pre- and post-synaptic neurones were based on the number of boutons supplied by one population and the number of possible targets (based on dendritic length) presented by the other – assuming no selectivity. Any part of any dendrite in a given layer, is assumed to be as likely to be innervated by an axon with boutons in that layer, as any other (excepting only the axonal initial segment targets of chandelier cells and somata being the exclusive domain of presynaptic basket cell axons).

Simplified circuits like these still form the basis for some models of the “canonical microcircuit” (e.g., Niell and Scanziani, 2021: Figure 1) despite evidence that some components are either extremely rare, or nonexistent, while others are more powerful than predicted. In particular, it is extremely difficult to find evidence for the interlayer, excitatory feedback loops, often popular with circuit modelers, which do not appear to exist in any functionally relevant form. Reciprocal innervation between spiny excitatory cells within layer 4, particularly those that both receive excitation from, or provide excitation to, another cell, is however, common enough to satisfy some models. Among the lowest hit rates reported are for those connections that might (if they existed) form within column excitatory-excitatory “back projections” from layer 3 to layer 4 and from layer 5 to layer 3 as well as for synapses made by layer 6 CT (corticothalamic) cell axons with other deep layer pyramidal cells, which preferentially innervate interneurones (Thomson, 2005 for review). In contrast, the highest “hit rate” reported for excitatory cell to excitatory cell connections is for the descending connections from layer 3 pyramidal cells to large layer 5, intrinsically burst-firing pyramids: higher than 1:2 if the two apical dendrites are adjacent. The large layer 5 cells rarely if ever return the favor, however (only one from a L5 spiny cell with unusual structure, of hundreds tested over many years).

Following this and similar studies it was for a while widely held that each excitatory connection involved only one synapse, so that all inputs had equal weight. It was also thought that the single synapse could be made anywhere on the postsynaptic dendritic tree within range. This idea, it seemed was preferable to having to consider how more selective innervation patterns might arise, or how this additional complexity might be modeled. However, comparisons of single axon EPSPs showed wide variations in time course and amplitude. Simply presenting data like these and proposing that it might result from additional complexity could not dispel these attractive notions, direct evidence was necessary.

To determine how many synapses actually contribute to a given connection and where on the postsynaptic dendritic tree they are, it was necessary to obtain good biocytin fills of both cells, and Avidin-HRP recovery of both – without using detergents to aid penetration of HRP through membranes. This allowed the putative synapses identified as close membrane appositions at the light level, to retain enough integrity to be confirmed at the EM level. At that time, this challenge was met by only a few particularly gifted and determined anatomists, Peter Somogyi and Jim Deuchars (e.g., Gulyás et al., 1993; Deuchars and Thomson, 1995; Thomson et al., 1995; Buhl et al., 1997; Tamás et al., 1997). These studies demonstrated that most, and in many cases all, of the close membrane appositions, between presynaptic axon and postsynaptic dendrite, had the ultrastructure expected of synapses. The following sequence of events is not unusual: one or two groups go to great lengths to satisfy both their own standards and the criticisms leveled when they produce data that conflicts with dogma. However, once this has been achieved, reviewers no longer demand the rigor expected before. In this case, it allowed other groups to dispense with demanding histological procedures and use detergents to make the processing easier. Apart from the loss of accurate information about axonal and dendritic diameters, now that historical archives are being discarded, perhaps the major loss here is that few young scientists see how beautiful peroxidase-labeled neurones really were before their lipid membranes were disrupted with detergents like Triton.

**Note: For those who have not followed the field, connections involving only one synapse, whether excitatory, or inhibitory, have been found to be rare when all of the relevant portions of the axonal and dendritic arbors are recovered.**

Studies that do not record and identify both of the neurones contributing to a synaptic connection also fail to capture finer detail. Are the synapses made by the axons of type A cells on type B cells distributed randomly, or restricted to certain postsynaptic compartments? Do they target basal or apical dendrites preferentially? Does it matter? How many synapses does a type A cell deliver to a type B cell and does the relative position of the two cells influence this number, or the position of the synapses? Are the physiological properties of each type of synaptic connection (classified by the class of pre- and postsynaptic neurones) unique? The simple answer to all of the above, so far as it has been studied, is yes (Thomson and Morris, 2002; Thomson and Lamy, 2007; Thomson, 2010 for reviews).

Recently, the technical advances that have allowed multiple neurones to be recorded simultaneously, have found similar patterns of selectivity (Perin et al., 2011; Jiang et al., 2015). To summarize: not only interneurones, but excitatory neurones are extremely selective, and not only in terms of which cells they will innervate, but where. For example, L4 spiny cell inputs to L3 (and L4) pyramidal cells terminate on relatively proximal portions of basal, but not on apical oblique dendrites. This despite considerable overlap in the three dimensional space occupied by the two types of dendrite. In contrast, layer 3 inputs to the same cells terminate more distally (97 *versus* 69μm) on both apical and basal dendrites (Feldmeyer et al., 2002, 2006; Bannister and Thomson, 2007; Spruston, 2008, for review). Moreover, in visual cortex, synapses from neurones with spatially overlapping receptive fields are more likely to be close neighbors on a layer 3 dendritic branch than chance would predict (Iacaruso et al., 2017). Though the preferred orientation of local inputs was not found to predict their spatial relationship (Chen et al., 2013; Cossell et al., 2015; Iacaruso et al., 2017) neighboring synapses from callosal and local axons did have similar orientation preferences (Lee et al., 2019).

Simultaneous activation of clustered inputs was found to be vital for the activation of voltage dependent events in layer 3 pyramidal dendrites. Near simultaneous EPSP-like events in no less than 7 or 8 closely neighboring dendritic spines (focal uncaging of RuBi-glutamate) was required to elicit all or none voltage-dependent events (Biró et al., 2018 for methods). Fast, probably Na^+^ spikes were activated in proximal dendritic compartments. At intermediate distances from the soma relatively fast, all or none, TTX insensitive (probably Ca^2+^) events were elicited. These were sometimes also activated by fast dendritic spikes, but were always terminated rapidly by a phrixotoxin-2-sensitive, AP-4-insensitive K^+^ conductance (probably Kv4.2, or Kv4.3). When distal sites were stimulated – slower, often more complex events were recorded from the soma. By their similarity to previous observations, these most probably resulted from the asynchronous activation of voltage dependent events in more than one fine terminal branch. Neither the all or none nature, time course, nor the shape of the proximal and intermediate, voltage-dependent events indicated a more significant contribution by NMDA-receptors than that of depolarizing the dendrite to threshold. Unfortunately, time and the unexpected absence of the PI at critical points prevented more detailed investigation of these interesting phenomena (Thomson unpublished, 2011–2015).

Although the synapses from any one presynaptic excitatory cell are located at similar electrotonic distances from the soma, they do not cluster, but distribute across multiple dendritic branches. Quantal EPSPs from a single axon therefore sum linearly at the soma: in layer 5 (Thomson et al., 1993), layer 4 (Bannister and Thomson, 2007), layer 6 (Mercer et al., 2005; West et al., 2006; Thomson, 2010), layers 3-6 (Brémaud et al., 2007), and in CA1 (Deuchars and Thomson, 1996). Moreover, none has a probability of release near one. The clustering of synapses from different presynaptic neurones with similar input preferences must increase, therefore, the chance that these voltage dependent events will be activated.

## A Prime Example of Hypothesis Driven Research, or the Dogged Pursuit of an Answer

We left the identification of central neurotransmitters when only cholinergic and adrenergic transmission were recognized. Although these systems have many important functions in the cns, by the 1950’s it had become clear that most fast information transfer was not mediated by either. The hunt was on for novel excitatory and inhibitory neurotransmitters – driven by the hypothesis that central synaptic transmission was mediated by as yet unidentified chemicals. However, before a new transmitter could be accepted by the scientific community a number of criteria – based on what was known of existing systems – had to be satisfied:

1. The substance should be present in the presynaptic neurones in question.
2. The presynaptic neurone should possess an enzymatic mechanism for its synthesis.
3. An enzyme that can render the substance inactive rapidly should be present.
4. The substance applied to the postsynaptic neurone should elicit the same response as the endogenous transmitter.
5. During stimulation the substance should be detectable in the extracellular fluid.
6. Pharmacological agents that interfere with synaptic transmission should affect the response to the exogenous substance, similarly.

Glutamate was found to be the most potent excitatory compound present in the crayfish nervous system (Kravitz et al., 1963b; Gerschenfeld, 1973), and spots that responded to iontophoretically applied glutamate coincided with the sites at which the natural transmitter acted (Takeuchi and Takeuchi, 1964). Moreover, glutamate had a selective uptake system (Iversen and Kravitz, 1968) which could replace for the inactivating enzyme of criterion 3. Similar studies presented γ-amino-butyric acid (GABA) as a strong candidate for an inhibitory transmitter in lobster (Kravitz et al., 1963a; [Bibr B114][Bibr B77], also Woodward, 1984).

In the late 1950’s, Curtis et al. (1959a,b, 1960, 1961) demonstrated that β-alanine, γ-amino-n-butyric acid (GABA) and glycine had depressant effects on spinal neurones, while their corresponding dicarboxylic acids, aspartic and glutamic acids, were powerfully excitatory (for review: Curtis and Watkins, 1965; Watkins and Evans, 1981).

Some of the excitatory and inhibitory amino acids identified by Jeff Watkins et al., were certainly present. The problem was not whether they were there, but that there was just too much of them, in all sorts of cells, involved in multiple metabolic pathways and with significant quantities in cerebrospinal fluid. They might be said to satisfy criteria 1 and 4, but their mere presence was not accepted as relevant to synaptic transmission. Moreover, no enzyme to destroy them could be identified at the synapse. In any case, how could such ordinary, ubiquitous, chemical compounds mediate such exquisitely precise transmission? To many it was unthinkable. In his excellent book, McLennan (1963) found their actions well worthy of mention, but only went as far as suggesting that they might have some humoral function in regulating the level of excitability in the c.n.s. Salmoiraghi et al. (1965) also appear open minded, in that they considered having to satisfy all six criteria at once, a tall order. They nevertheless list their own three criteria, which approximate to criteria 1, 2 plus 3, 4 plus 6, and 5, above.

Even as late as the early 1970’s doubters probably still outnumbered adherents. In an otherwise excellent text book recommended to physiology students (including the author of this piece), Aidley (1971) wrote:

‘Curtis et al. (1960) *found that glutamic, aspartic and cysteic acids cause excitation of spinal interneurones and motoneurones …Again, however, this seems to be a non-specific response, so that none of these acids can be regarded as transmitter substances… The physiological significance of the effects of glutamate and GABA, which occur in quite high concentrations in parts of the nervous system, is obscure.*’

Jeff Watkins (2006) later described how he came to work with David Curtis: ‘*… with a PhD in chemistry, I faced a big problem – what actually to do for the rest of my life…. It struck me with immense force that thought, feelings and* ‘*instructions for behaviour’ were all generated within a mass of pinkish grey gelatinous substance inside our heads, made entirely of chemicals!!* [at Yale] *One of my American friends… suggested that I get in touch with a renowned Australian compatriot of mine, J.C. Eccles. I did. Professor Eccles was most enthusiastic about the prospect of a chemist joining his physiological laboratory in Canberra and promptly offered me a job as a Research Fellow to work with David Curtis, on chemical transmitters in the brain. Interestingly, Eccles himself had only just begun to believe in them, but had now become a most enthusiastic convert from his long-held previous conviction that all central synaptic transmission was electrical. At the age of 26 my future direction in life was established*.’

By the structure-activity relations of those compounds that excited neurones, those that inhibited, and those that blocked these actions competitively, it became clear that there was more than one type of EAA receptor. By the late 1970s three glutamate receptor types had acquired the names of their most selective known agonists: NMDA (N-methyl-D-aspartate), Quisqualate^∗^ and Kainate-receptors.

**^∗^Note: The quisqualate receceptor was renamed when a more selective agonist α-amino-3-hydroxy-5-methyl-4-isoxazolepropionic acid (AMPA) was synthesized by Tage Honoré (Honoré et al., 1982; Watkins et al., 1990a, b), AMPA receptor, or AMPAR, and later still GluR.**

This simple division of ligand-gated glutamate receptor-channels (ionotropic receptors) into three distinct types was – as we will see – more than enough for many to deal with in the 1980s, when we did not know that within the decade things were to become a lot more complicated. When the technical expertise of Peter Seeburg was combined with Hannah Monyer’s imagination and clinical insight, these receptors were found to exist in different forms, depending on the subunits they contained and which splice variants were expressed; a diversity that correlated with brain region and developmental stage (Sommer et al., 1992; Monyer et al., 1994, for reviews). Nor was this the end of the story; soon metabotropic glutamate receptors (mGluR’s) with different coupling mechanisms and their own pharmacology entered the stage (Schoepp and Conn, 1993; Pin and Duvoisin, 1995, for review). But, to return to the simpler world of the 1980s.

There had been interest in the possible clinical relevance of the NMDA receptor since the dissociative anesthetics ketamine (“Big K”) and phencyclidine (PCP or “Angel Dust”) were found to block NMDA receptors selectively (Anis et al., 1983). These drugs induce anesthesia and a potent central analgesia, as well as hallucinations (Canadian Medical Association, 1969), and persecutory ideation (Domino and Luby, 1981; Jasinski et al., 1981; Caracci et al., 1983; Javitt and Zukin, 1991). However, NMDA receptors became truly fashionable when Stephen Kehl demonstrated that the selective NMDA receptor antagonist, DL-2-amino- 5- phosphonovalerate (APV, or AP-5) prevented long term potentiation (LTP) of the field potential elicited by high frequency stimulation of the Schaffer collateral-commissural pathway (Collingridge et al., 1983). However, they – like everyone else who had tried thus far – failed to demonstrate even partial block by APV of an EPSP in the mammalian cns. Despite the effects of admittedly less selective antagonists on spinal reflex pathways in the frog and cat (Watkins and Evans, 1981, for review), many influential scientists remained convinced that NMDA receptors were only involved in unnatural or pathological activity – not in normal synaptic transmission.

Whether or not these receptors had a physiological role, they did have unusual and interesting characteristics which attracted attention. Responses to NMDA increased when [Mg^2+^] was reduced below physiological concentrations^∗^ (Davies and Watkins, 1977; Evans et al., 1977; Ault et al., 1980; Scatton and Lehmann, 1982), or when the neuronal membrane was depolarized from rest (MacDonald et al., 1982; Dingledine, 1983; Flatman et al., 1983).

**^∗^Note: Basic “Ringer’s solution” – as often used in amphibian preparations – does not contain magnesium.**

## Chance, Serendipity and the NMDA Receptor

The causal link between magnesium block and an unusual voltage-relation only became apparent when two labs simultaneously, though quite independently, discovered that Mg^2+^ blocks the NMDA receptor channel in a voltage dependent manner (Mayer et al., 1984; Nowak et al., 1984). Mayer and Westbrook (1984b) had already shown that the anomalous response of cultured spinal neurones to glutamate was due to the involvement of two conductances. One showed a conventional voltage relation, the other – which was blocked by APV – was voltage-sensitive. To investigate the ions involved in this unusual conductance was a logical next step.

Not so the parallel discovery in Philippe Ascher’s lab. The Parisian story has more to do with serendipity than with any specific idea or plan, as Philippe Ascher explained (Ascher, 2014):

‘*With Linda Nowak, the new postdoc who had arrived from Ann Arbor, we decided to try primary cultures of mice cortical neurons. We had no hood, no incubators, and, for a whole year, we obtained the cultures from laboratories within walking distance—those of Jeanine Koenig or Alain Prochiantz.* ***We wanted to see single channels opened by neurotransmitters, and I think that we selected glutamate because it was the cheapest drug to start with***. *For many weeks we did not see any response, until I remembered an article in which Jeff Watkins et al., had described a “non-competitive” effect of magnesium ions (Mg^2+^) on the glutamate receptors selectively activated by N-methyl-D-aspartate (NMDA receptors). We prepared a Mg^2+^-free ringer and readily observed that glutamate induced a large noisy current in the whole-cell mode and the expected single channel currents in outside-out patches. Adding Mg^2+^ reduced the whole cell current and introduced a negative resistance around resting potential.*’

In 1984, I was merely a bystander in the EAA field when Tony Angel sent his Ph.D. student, Chris Pollard, to learn how I made intracellular recordings from in brain slices (Angel and Pollard, 1985). Chris wanted to record from neocortex rather than hypothalamus, where I was working, so we had a go. Cortical pyramidal cells were much easier to record than suprachiasmatic neurones and became the test system for my rig. I became intrigued by some of the EPSPs elicited in cortical pyramidal cells by “minimal stimulation” of the underlying white matter, which exhibited an unconventional voltage relation and produced an apparent increase in input resistance. I might have stopped there, but by happenchance I had heard Mark Mayer present his recent findings with Gary Westbrook to the Physiological Society (Mayer and Westbrook, 1984a). I checked the abstract and began to suspect that these EPSPs might be mediated by NMDA receptors. A testable hypothesis had been provided by chance, and since I knew the groups in London studying EAA pharmacology, the necessary agonists and antagonists were available. The resultant submission (Thomson et al., 1985; also Thomson, 1986) received two extremely brief reviews, both insisting on rejection. One stated that it was obvious that NMDA receptors would be activated by synaptic glutamate release and the results were not therefore novel. The other wrote: “*These results go against the firmly held convictions of most of the people in this field*” and should not, therefore, be published. That study might have ended there, but our Nature editor enclosed a brief note with the referees’ reports: ‘*The referees’ comments are enclosed for your amusement. The paper is accepted for publication*.

Just as EM level confirmation of multiple synapses from a single axon was required to refute the firmly held convictions of many in the field, painstaking iontophoretic controls were required to convince others that the concentrations of NMDA receptor antagonists used in this study could be considered selective at synapses – thereby, apparently, removing the requirement for other labs to repeat these controls.

A few years later, another serendipitous discovery was made in Philippe Ascher’s lab (Johnson and Ascher, 1987, 1992). Neurones and patches responded much more vigorously to NMDA in medium that had been “conditioned” by contact with the neuronal culture. The conditioned medium – they surmised – must contain something that augments responses to NMDA. It turned out to be glycine, acting at a strychnine-insensitive co-agonist-site. Both glutamate and glycine (or suitable analogs thereof) must bind to their respective sites in the NMDA receptor before it can open (Henderson et al., 1990). At first, since the glycine concentrations measured in brain are far higher than those required to saturate it, this glycine site was thought to be permanently saturated and not therefore suitable as a therapeutic target. However, iontophoretically applied glycine was found to augment NMDA receptor mediated EPSPs in brain slices (Thomson et al., 1989), responses to EAAs acting at NMDA receptors in the thalamus *in vivo* (Salt, 1989) and in neocortical slices *in vitro* (Thomson, 1990a, b). Since activity at this glycine site can be modified by a range of partial agonists as well as by manipulating glycine and D-serine transporters (GlyT1 and arginine-serine-cysteine transporter-1, Asc-1, respectively), these studies have triggered interest in therapies for conditions ranging from schizophrenia to post traumatic stress disorder.

A simple explanation for the apparent lack of NMDA receptor involvement in the Schaffer collateral, commissural pathway during low frequency stimulation, while high frequency stimulation induces NMDA receptor-dependent LTP, only became apparent when synaptic connections between CA1 pyramidal cells were found to have a strong NMDA receptor mediated component. The axons of CA1 pyramids are confined to *stratum oriens* and *alveus* as are the CA1 pyramid-pyramid synapses on basal dendrites, and are not activated by electrical stimulation in *stratum radiatum* – unless CA1 pyramidal cells fire. Coactivating these synapses with minimal Schaffer EPSPs resulted in lasting enhancement of the latter (Radpour and Thomson, 1991; Thomson and Radpour, 1991; Deuchars and Thomson, 1996).

## Serendipity Has Also Led to Technical Advances

For the questions about receptor-channels they wanted to answer, Erwin Neher and Bert Sakmann had very good reason to develop patch clamp techniques and knew theoretically what would be required for single ion channel openings to be recorded faithfully. For 5 years, however, they did not succeed until chance intervened:

*“We made many systematic attempts to overcome the seal problem (manipulating and cleaning cell surfaces, coating pipette surfaces, and reversing charges on the glass surface, etc.) with little success… By about 1980, we had almost given up on attempts to improve the seal, when we noticed by chance, that the seal suddenly increased by more than two orders of magnitude when slight suction was applied to the pipette. The resulting seal was in the gigaohm range, the so-called* “*Gigaseal*”… And the rest, as they say, is history.

Neher does, however, point out one of the limitations of the technique:

“*The cell-attached measurement… leaves the cell largely intact, and allows one to observe channels open and close, or to record action potentials extracellularly… Excised patches constitute the other extreme, where membrane patches are removed from their natural environment for optimal control of solution composition on both sides of the membrane. The whole-cell recording method is at an intermediate position in this respect. It does provide excellent control over membrane potential, if cells smaller than 20 μm in diameter are used. However, the chemical composition of the internal medium is neither undisturbed, nor is it under good control. We found that small mobile ions typically exchange by diffusion between pipette and cell in a few seconds… Molecules of intermediate size, like second messengers, typically* “*wash-out” or* “*load” into cells within 10 seconds to a minute, and small regulatory proteins may take several minutes and longer for complete equilibrium*.” (Neher, 1991).

Whole cell electrodes made discontinuous single-electrode voltage clamp of neurones feasible and for a time EPSCs and IPSCs were all the rage, EPSPs and IPSPs were passé, despite the well-earned reverence for Wilfred (Wil) Rall’s pioneering application of cable theory to dendrites (Rall, 1959). In time, however, it began to be understood, not only that rapid changes in voltage in long dendrites could not be adequately clamped, but that neurones do not operate in current, but in voltage mode. Neuronal modelers must have sighed with relief when they no longer had to add to their model the properties and limitations of the discontinuous clamp as well as those of the neurone, in all its complexity.

## Read the Methods Section

Infrared, differential interference contrast microscopy enhances visualization of somata and proximal dendrites in brain slices, allowing whole cell pipettes to be positioned on selected neurones. The introduction of mouse lines in which neurones are fluorescently labeled according to the expression of specific markers allows labeled neurones to be identified *in vitro* (and indeed, in the superficial neocortical layers *in vivo*). These approaches make recordings from single cells, pairs and groups of neurones much easier, especially for the novice, and the low resistance of the pipettes reduces noise.

However, this approach requires the slices to be submerged, which in turn typically requires thinner slices (≤300 μm thick) to be obtained from juvenile rodents (though see Jiang et al., 2015) and recorded at temperatures close to 20°C. Comparison of cell and synaptic properties recorded under these conditions and those obtained from 450-500μm thick slices obtained from adult rats (and cats), and recorded in an interface chamber at 35°C, showed that the basic properties of each type of cell and synapse studied were broadly comparable in the two cases, but that every voltage-gated and synaptic event was close to four times slower in cool, juvenile slices (Ali et al., 2007). Be careful, therefore, to read the Methods sections thoroughly (even if they are relegated to a supplement, or appear in another article) before combining data from more than one study in your model.

## Simplification Driven by Common Practice and Technology

A number of simplifications have arisen from the modern habit of applying rigid protocols. For example it is now commonplace for a synaptic connection to be labeled “depressing,” or “facilitating,” on the basis of responses to presynaptic action potentials at a single, fixed interspike interval. Moreover there are many who think that they know what these results mean and merrily extrapolate from incomplete information. The use of such protocols can perhaps be forgiven when the question is simple, such as who connects with whom? For example, when 8 neurones communicating through anything up to 56 possible connections (64 if autapses are considered) are recorded simultaneously. All the resultant connections have to be tested, along with the electrophysiological characteristics of each neurone (with another fixed protocol). There is no time in which to investigate any cell or connection in the detail required to reveal the exquisitely fine tuning of information transfer that differs from one type of synapse to another (Thomson, 2000; see also Südhof, 2014, 2018 if you enjoy complexity).

Even more importantly perhaps, protocols like these stifle exploration and adventure – little that is unexpected is likely to be found, because you can, as Popper warned, only find what you look for. I appreciate that PIs are anxious to obtain as much “reliable” and repeatable data as the next quality article requires, while inexperienced experimentalists may be too nervous to try something that has not been tried and tested, or that might contradict dogma. Some of the most productive electrophysiological labs are in danger of becoming factories, designed to generate what the “field” expects, with humans relegated to prescribed tasks that might soon be within the capabilities of an automaton. One can, however, hope that when the requisite information has been saved, the more adventurous will be encouraged to play, to find out, “What happens if…?” Neurophysiologists should not leave all the adventures to biochemists.

A V Hill also looked into the future for young scientists with concern. His department had flourished when science was inexpensive and much equipment was built in house, but he foresaw the time when sums out of the reach of those without substantial external funding would be needed. It was still possible for young scientists to strike out independently in the 1980’s, if they were prepared to build, adapt or at least design, most of the equipment themselves. Universities still had excellent electronic and mechanical workshops and many scientists had a wide range of manual, as well as intellectual skills. Back then, even an “off the shelf,” deluxe electrophysiological rig might have cost a few thousand pounds. State of the art now runs into hundreds of thousands, not to mention the cost of maintaining mouse lines, or the reagents needed for cutting edge biochemistry. How many young scientists will be able to use the skills gained in a well-funded, state of the art lab, when – if ever – they attempt to strike out on their own? We may think we are helping our students and postdocs by providing them with luxuries we never had, building labs in which it is easier for them to obtain the data for high impact publications – but are we?

## Translation

In addition to the appalling concept that scientists should compete (the dreadful image of some future reality television show springs to mind), “translation” has entered the jargon associated with funding. As it becomes more difficult for the awarding agencies to justify the increasingly expensive “basic” research they fund, if they cannot claim that at least some of it will drive new commercial, technical, medical or societal advances; aspiring applicants are encouraged to provide evidence that their project will “translate” into something useful. Are we not allowed to do it because it is fun anymore, or because we want to test some crazy idea. Meanwhile, “Big Pharma” has found that the research it was doing in neuroscience just does not pay, and has cut much of the funding in this area, leaving future adventures in the treatment of many neurological disorders to venture capital. Is it still practical to think as AV Hill did?

“*With faith in the ultimate usefulness of all real knowledge a man may proceed to devote himself to a study of first causes without apology, and without hope of immediate return.*”

## Summary

Hypotheses used as summaries of what we know – or think we know – can be useful and some significant advances have been made by trying to refute a hypothesis – usually one proposed by someone else. It is also the case that time has been wasted and dead ends reached in attempting to support, or comply with, an inadequate hypothesis. What much of the foregoing illustrates is that in many cases, we would not have reached the point at which an important, testable hypothesis could be proposed unless someone had made a chance observation, or collected a butterfly; one that they did not ignore as an artifact, or choose not to pursue because it contradicted current ideas. These observations are made and developed when and where the right environment exists, where exploration is encouraged and where we can question any idea that is becoming widely accepted as an inevitable truth; it can become dangerous.

## Author Contributions

The author confirms being the sole contributor of this work and has approved it for publication.

## Conflict of Interest

The author declares that the research was conducted in the absence of any commercial or financial relationships that could be construed as a potential conflict of interest.

## Publisher’s Note

All claims expressed in this article are solely those of the authors and do not necessarily represent those of their affiliated organizations, or those of the publisher, the editors and the reviewers. Any product that may be evaluated in this article, or claim that may be made by its manufacturer, is not guaranteed or endorsed by the publisher.
